# Prevalence and risk factors for postoperative stress-related cardiomyopathy in adults

**DOI:** 10.1371/journal.pone.0190065

**Published:** 2017-12-20

**Authors:** Tak Kyu Oh, In-Ae Song, Young-mi Park, Jung-Won Hwang, Young-Tae Jeon, Sang-Hwan Do, Yeonyee E. Yoon, Soyeon Ahn, Jae-sung Lee

**Affiliations:** 1 Interdepartment of Critical Care Medicine, Seoul National University Bundang Hospital, Bundang-gu, Seongnam-si, Gyeonggi-do, Korea; 2 Department of Anesthesiology and Pain Medicine, Seoul National University Bundang Hospital, Bundang-gu, Seongnam-si, Gyeonggi-do, Korea; 3 Medical Research Collaborating Center, Seoul National University Bundang Hospital, Bundang-gu, Seongnam-si, Gyeonggi-do, Korea; 4 Department of Cardiology, Cardiovascular Center, Seoul National University Bundang Hospital, Bundang-gu, Seongnam-si, Gyeonggi-do, Korea; University of Messina, ITALY

## Abstract

Stress-related cardiomyopathy can develop during the postoperative period due to surgery-related stress factors. However, the prevalence and risk factors for this condition are not yet known. During a retrospective, observational study, patients older than 19 years who underwent procedures from January 2011 to December 2015 at a tertiary hospital were included. The main aim was to identify the prevalence and related risk factors for postoperative stress-related cardiomyopathy. To estimate the incidence per risk factor, univariate and multivariate Poisson regression analyses were performed. During the 5-year period, 95,840 patients older than 19 years underwent 125,314 procedures, and the prevalence of postoperative stress-related cardiomyopathy was 17.74 per 100,000 (95% confidence interval, 9.31–26.17), with an in-hospital mortality of 23.5%. As a result, three risk factors were significantly associated: preoperative American Society of Anesthesiologists classification (incidence rate ratio, 5.901 for American Society of Anesthesiologists class 1–2 [ref] versus 3–6; 95% confidence interval,1.289–27.002; *P* = 0.022); preoperative body mass index (incidence rate ratio, 1.247 for increases of 18.5 [ref] to 30; 95% confidence interval, 1.067–1.458; *P* = 0.006); and preoperative serum sodium (incidence rate ratio, 0.830 for each increase of 10 mmol/L from 130; 95% confidence interval, 0.731–0.942; *P* = 0.004). The incidence rate ratio for age for each increase of 10 years from 50 years was 1.057, but it was not statistically significant (*P* = 0.064). Our study found that the prevalence of postoperative stress-related cardiomyopathy was 17.74 patients per 100,000 adult patients over the course of 5 years, with four cases of in-hospital mortality. Factors that increased the risk of postoperative stress-related cardiomyopathy included higher American Society of Anesthesiologists class (≥3), preoperative hyponatremia, and higher preoperative body mass index.

## Introduction

Stress-related cardiomyopathy (SRC) is a disease that was first reported in Japan in the early 1990s [1]. It is also referred to as Takotsubo cardiomyopathy (TTC), and it is known to be related to reversible cardiac dysfunction with stressful conditions [1]. The mechanism for the development of SRC is hypothesized to be sympathetic stimulation of the myocardium of the heart associated with increased catecholamine [2, 3], and it mostly develops in postmenopausal women and patients with neurologic injuries [4, 5]. Additionally, all conditions causing stress-like sepsis [6], epileptic seizure [7], opioid withdrawal [8], pheochromocytoma [9], and emotional stress [10] are known to precipitate SRC.

Surgery causes great physiologic and psychological stress for patients [11, 12]. Furthermore, patients are exposed to various complications such as postoperative infections [13]. In addition, postoperative pain disrupts the emotional stability of patients and can increase sympathetic tone [14]. Therefore, SRC can develop during the postoperative period [15]. In fact, some case reports have reported the occurrence of postoperative SRC [16, 17]. However, studies of the prevalence of and risk factors for SRC during the postoperative period and involving a large population have not been conducted. One study in 2010 reported 17 cases of procedure-related SRC over the course of 63 months; however, the focus of this study was on the outcome of SRC and clinical presentation rather than on the prevalence and risk factors [18]. Therefore, additional research regarding the prevalence and risk factors for postoperative SRC is needed. The purpose of this study was to investigate the prevalence of and risk factors related to postoperative SRC.

## Materials and methods

This retrospective, observational study was approved by the Institution Review Board of Seoul National University Bundang Hospital (SNUBH) (approval number: B-1704/393-107). Due to the retrospective design, informed consent was not required. For this study, the medical records of adult patients at least 19 years old who underwent surgery or other procedure in the operating room of SNUBH from January 1, 2011 to December 31, 2015 were collected. If a patient underwent two or more surgeries or other procedures, then only the last surgery or procedure was reflected in the analysis; inaccurate medical records were excluded from the analysis. As of July 2017, SNUBH has been a 1,360-bed tertiary hospital with 38 operating suites where, on average, 150 elective or emergency procedures are performed daily. In addition, the electronic medical record system has been used since 2003 to manage and archive all medical records accurately.

### Definition and diagnosis of postoperative SRC

To diagnose postoperative SRC, characteristics of the postoperative setting were considered; additionally, the Johns Hopkins criteria for the diagnosis of SRC were set as the standard for this study [19]. The diagnostic method for SRC recently reported by a study conducted at SNUBH was referred to as a reference [20]. To diagnose postoperative SRC, the following criteria had to be satisfied: 1) among patients who did not have symptoms that may indicate preoperative SRC (e.g., chest pain, change on the electrocardiogram, shock, hypoxia, altered mentality, and dyspnea), SRC developed before discharge; 2) findings indicating SRC (e.g., typical apical ballooning and ventricular wall motion abnormality) detected using a two-dimensional (2D) echocardiographic evaluation after the observation of symptoms; 3) lack of significant obstruction greater than 75% in the coronary artery on coronary angiogram; and 4) recovery of ventricular wall motion abnormality within days or weeks. As a result, postoperative SRC was diagnosed when criteria numbers 1, 2, 3, and 4 were satisfied. Preoperatively, 2D echocardiography was conducted. Patients with pre-existing regional wall motion abnormality or the presence of dilated, hypertrophic, or restrictive cardiomyopathy were excluded. Generally, at SNUBH, an evaluation to determine SRC is performed through cardiologic consultation if ischemic heart disease is suspected during the postoperative period [19].

SRC was diagnosed during the study period using bedside 2D echocardiography after consultation with the cardiologist and after coronary evaluation. For study enrollment, the Medical Informatics Team screened the patients who were diagnosed with SRC. Then, two certified intensivists (T.K. Oh and I.A. Song) confirmed that the aforementioned diagnostic criteria were met. Finally, patients with postoperative SRC were selected for study inclusion.

### Data collection

For this study, the following data were collected through the SNUBH Pre-Anesthetic Record Registry and Surgery Record Registry: demographic data (body mass index [BMI], age, and sex), preoperative laboratory test results (hemoglobin level [g/dL], white blood cell count [×1000/μL], platelet count (×1000/μL), prothrombin time-to-international normalized ratio, aspartate aminotransferase level [IU/L], alanine aminotransferase level [IU/L], albumin level [g/dL], glucose level [mg/dL], activated partial thromboplastin time [seconds], blood urea nitrogen [mg/dL], creatinine level [mg/dL], serum sodium level [mmol/L], and serum potassium level [mmol/L]), official preoperative electrocardiogram findings, airway status, presence of hypertension, presence of diabetes, history of preoperative ischemic heart disease, history of preoperative neurologic disease, American Society of Anesthesiologists (ASA) classification, history of surgery, type of anesthesia, postoperative intensive care unit (ICU) admission, and nine types of surgeries (cardiovascular; thoracic; general; neurosurgical; orthopedic; urologic or obstetrics and gynecologic; ear, nose, and throat; plastic; and dental and eye with or without sedation). The results of each preoperative laboratory test were the latest results before the procedure. During airway evaluation, cases with Cormack grades III and IV were considered difficult; a history of preoperative ischemic heart disease including stable angina and myocardial infarction and intracranial hemorrhage immediately preoperatively were included as preoperative neurologic diseases. The primary outcome of this study was to identify the prevalence of postoperative SRC for 100,000 patients during the 5-year study period.

### Statistical analysis

We explored the risk factors expected to contribute to the development of SRC using Poisson regression, which is known to be suitable for modeling event data for rare occurrences. For some categorical variables, the significance of risk factors was tested by Firth-corrected Poisson regression to reduce the bias of estimation. First, univariate Poisson regression was used to calculate the crude prevalence of SRC by substituting each category of risk factor with *P*≤0.05 in the estimated Poisson regression equation. Continuous variables were divided into three to four categories within the normal range of clinically significant, and the categorical variables were used for regression substitution as originally classified. The calculated crude prevalence of SRC was adjusted to prevalence per 100,000 patients and presented in the results. Finally, we used forward stepwise multivariate Poisson regression analysis was performed to investigate how the risk of SRC changes according to independent risk factors. The risk factors, which were risk factors for postoperative SRC during univariate Poisson regression (*P*<0.05), were included in the multivariate Poisson regression analysis. The results of multivariate Poisson regression analysis were shown as the incidence rate ratio (IRR) and as the prevalence per 100,000 patients. Statistical analyses were performed and graphics were generated using the open-source statistical software R (version 3.3.2; http://www.R-project.org) *P*<0.05 was considered statistically significant.

## Results

At SNUBH during the study period, 125,314 surgeries were conducted for 95,840 patients (53,626 [56%] female patients) older than 19 years. Among them, 17 patients were diagnosed with postoperative SRC. S1 Table shows information for patients who developed postoperative SRC. Hospital mortality occurred for four patients (23.5%). Sepsis developed during the postoperative period in five patients (29.4%).

### Prevalence of postoperative SRC according to univariate poisson analysis

**Fig 1:**
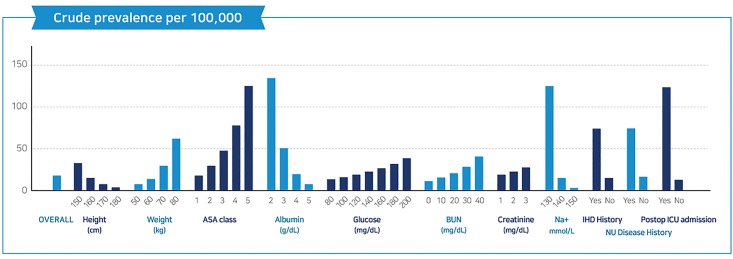
Crude prevalence per 100,000 postoperative stress-related cardiomyopathies over the course of 5 years. ASA, American Society of Anesthesiologists; BUN, blood urea nitrogen; ICU, intensive care unit; IHD, ischemic heart disease; NU disease, neurologic disease; postop, postoperative.

Table 1 and Fig 1 show the simple incidence of postoperative SRC for each risk factor. Not considering the risk factors, postoperative SRC occurred in 17.74 patients per 100,000 patients (overall predicted prevalence: 17.74 per 100,000; 95% confidence interval [CI], 9.31–26.17). There was no significant sex difference in the incidence of postoperative SRC (female-to-male ratio, 10/17; 58.8%) Furthermore, preoperative risk factors that induced a significant difference in the prevalence of SRC were age, glucose level, blood urea nitrogen level, creatinine level, and preoperative ASA class; an increase in these factors was correlated with an increase in the prevalence of postoperative SRC (*P*<0.05). Patients with a history of preoperative ischemic heart disease (predicted prevalence, 73.76 per 100,000 patients; 95% CI, 1.50–146.02) and preoperative neurologic disease (predicted prevalence: 74.50 per 100,000 patients; 95% CI, -9.77–157.77) had an increased prevalence of postoperative SRC. In addition, patients who were admitted to the ICU postoperatively showed a higher prevalence of postoperative SRC (predicted prevalence: 123.92 per 100,000 patients; 95% CI, 15.37–232.46).

**Table 1:** Incidence of postoperative stress related cardiomyopathy in a single tertiary center for five years using univariate poisson regression analysis.

Risk factor		Predicted Prevalence per 100,000 individuals			
		Estimate (n)	lower CI	Upper CI	*P*-value
ALL		**17.74**	9.31	26.17	
Age	50	6.58	0.88	12.28	<0.001
	60	13.85	5.48	22.23	
	70	29.16	15.28	43.03	
	80	61.38	25.93	96.82	
Preop PT INR	1	18.14	9.52	26.77	0.001
	2	29.37	13.21	45.53	
	3	47.53	12.35	82.72	
	4	76.93	1.50	152.37	
Preop Albumin (g/dL)	2	134.37	-36.33	305.06	0.001
	3	50.83	11.47	90.19	
	4	19.23	9.15	29.30	
	5	7.27	1.46	13.09	
Preop serum Glucose (mg/dL)	80	12.84	6.61	19.08	<0.001
	100	15.37	8.00	22.75	
	120	18.39	9.63	27.16	
	140	22.01	11.55	32.48	
	160	26.34	13.79	38.89	
	180	31.52	16.40	46.65	
	200	37.72	19.41	56.03	
Preop BUN (mg/dl)	0	10.29	5.14	15.43	<0.001
	10	14.51	7.51	21.52	
	20	20.48	10.74	30.22	
	30	28.90	15.01	42.80	
	40	40.79	20.51	61.07	
Preop serum Creatinine (mg/dl)	1	18.39	9.63	27.15	0.001
	2	22.44	11.60	33.29	
	3	27.39	13.24	41.55	
Preop serum Sodium (mmol/L)	130	124.76	48.39	201.13	<0.001
	140	14.62	7.16	22.08	
	150	1.71	0.18	3.24	
History of Ischemic Heart Disease	Yes	73.76	1.50	146.02	0.04
	No	14.38	6.56	22.19	
History of Neurologic Disease	Yes	74.50	-9.77	158.77	0.013
	No	15.25	7.26	23.24	
Preop ASA classification	1	3.38	-2.02	8.78	ref.
	2	25.49	10.76	40.22	0.022
	3	80.78	6.14	155.41	0.002
	4	568.18	-341.10	1477.46	0.000
	5	1315.79	-2331.39	4962.97	0.000
	6	2173.91	-3851.87	8199.69	0.000
Postop ICU admission	Yes	123.92	15.37	232.46	0.025
	No	13.07	5.68	20.47	

CI, Confidence Interval; Preop, Preoperative; PT INR, Prothrombin Time international normalized ratio; BUN, Blood Urea Nitrogen; ASA, American Society of Anesthesiologists; Postop, Postoperative ICU, Intensive Care Unit

### Independent preoperative risk factors for postoperative SRC according to multivariate poisson analysis

Table 2 shows the predicted prevalence per 100,000 and IRR for each risk factor with *P*<0.1 according to the multivariate Poisson regression analysis. As a result, three risk factors were significantly associated: preoperative ASA classification (IRR, 5.901 for ASA 1–2 [ref] versus 3–6; 95% CI, 1.289–27.002; *P* = 0.022); preoperative BMI (IRR, 1.247 for increases of 18.5 [ref] to 30; 95% CI, 1.067–1.458; *P* = 0.006); and preoperative serum sodium (IRR, 0.830 for each increase of 10 mmol/L from 130; 95% CI, 0.731–0.942; *P* = 0.004). IRR for age for each increase of 10 years from age 50 years was 1.057, but this was not statistically significant (*P* = 0.064)

**Table 2:** Final risk factors for occurrence of postoperative SICMP using multivariate stepwise poisson regression analysis.

Risk factor		Predicted Prevalence per 100,000 individuals		
		Estimate (n)	95% lower CI	95% Upper CI
ALL		**17.74**	9.31	26.17
Age	50	5.34	-1.72	12.40
	60	9.27	0.00	17.55
	70	16.10	4.46	27.74
	80	27.95	1.02	54.88
Preop ASA classification	1–2	7.08	0.00	14.04
	3–6	41.76	-4.88	88.4
Preop Body Mass Index	18.5	3.04	-1.34	7.43
	23	8.22	0.83	15.61
	25	12.79	3.21	22.36
	27	19.89	5.7	34.08
	30	38.57	4.13	73.02
Preop serum Sodium	130	56.86	-6.53	120.25
	140	8.79	1.43	16.15
	150	1.36	-1.14	3.86
	Multivariate Poisson Regression			
	IRR*	95% lower CI	95% Upper CI	*P*-value
Age^a^	1.057	0.997	1.120	0.064
Preop ASA classification^b^	5.901	1.289	27.002	0.022
Preop Body Mass Index^c^	1.247	1.067	1.458	0.006
Preop serum Sodium^d^	0.830	0.731	0.942	0.004

IRR of Age^a^: increasing 10 years old from 50 years old,

Preop ASA classification^b^: 1–2 (ref) versus 3–6,

Preop Body Mass Index^c^: increasing by 18.5 (ref)-23-25-27-30,

Preop serum sodium^d^: increasing 10 mmol/L from 130

IRR*, Incidence Rate Ratio

CI, Confidence Interval; ASA, American Society of Anesthesiologists

## Discussion

Our study is the first to report that the estimated prevalence of SRC during the postoperative period is 17.74 patients per 100,000 adult patients. This number is slightly lower than the prevalence of SRC reported by the 2008 Nationwide Inpatient Sample Database of the United States (19.06 patients per 100,000 hospitalized patients) [21]. On the basis of these data, we found that preoperative BMI, ASA class, and serum sodium level were risk factors associated with the prevalence of postoperative SRC.

The important point of our study, which was different from that of other studies, was that sex did not contribute to the development of postoperative SRC. In fact, among 17 patients with postoperative SRC, the female-to-male ratio was 10:17 (58.8%), thereby showing a difference from other studies that reported that 80–90% of SRC patients were women [22]. For such a result, three factors must be considered. First, sex could have had an effect on the postoperative setting. According to a previous study [23], regarding the prevalence of SRC, physical stress has a greater effect on men and emotional stress has a greater effect on women. Considering that physical stress has a higher contribution during the postoperative period, there is a possibility that the prevalence of SRC is higher for men. A previous prospective study of patients with left ventricular apical ballooning due to physical stress indicated that the male-to-female ratio was 65:92 (70.7%) [24]. Additionally, the statistical issue is important. In contrast to previous reports [22], our study used total cohorts of a population of more than 95,000 to perform univariate and multivariate Poisson regression analyses. Considering proportion, approximately 56% of the total cohorts in our study were female. However, female sex was not an independent risk factor in our Poisson regression analysis, even though there were more female postoperative SRC patients (58.8%; 10/17) If another study used a similar statistical method to investigate independent risk factors, then the results could be different. Finally, we defined postoperative SRC not only as apical typical ballooning but as a ventricular wall motion abnormality. In our study, 8 of the 11 patients who had typical apical ballooning on 2D echocardiography were female. If we defined postoperative SRC as only typical apical ballooning on 2D echocardiography, as for TTC, then female sex could be a risk factor. However, additional studies of the effects of sex on the development of postoperative SRC are needed.

Another interesting point about our study included the newly identified risk factors for postoperative SRC. Older age and history of preoperative ischemic heart disease or neurologic disease have been reported to be associated with the incidence of SRC [5, 25]. However, these factors were not independent risk factors in our multivariate Poisson regression analysis. Instead, higher ASA class (3–6), which reflects overall comorbidities, was an independent risk factor in our study. Higher preoperative BMI was identified as an independent risk factor for postoperative SRC in this study. Obesity or higher BMI is a well-known risk factor for cardiovascular disease [26], and our study showed that it also could be applied for postoperative SRC. Finally, preoperative hyponatremia was a significant risk factor in our study. There were some reports that hyponatremia is associated with precipitation of SRC [27, 28]. Although there is controversy regarding this pathophysiology of hyponatremia and SRC, some assumptions have been suggested. First, preoperative hyponatremia was associated with preoperative comorbidities such as liver cirrhosis, nephrotic syndrome, and syndromes of inappropriate antidiuretic hormones and endocrinopathy [29]. These preoperative comorbidities might precipitate postoperative SRC. Second, hyponatremia could cause intracellular calcium overload due to the impaired function of the myocyte membrane sodium–calcium pump. However, additional studies are needed to clarify the effect of hyponatremia on postoperative SRC [30].

Another important point to consider in our study is that neuro-stress-induced cardiomyopathy could have overlapped postoperative SRC in our study. Andò et al. [31] and Trio et al. [32] reported that SRC could occur with neurovascular events (e.g., subarachnoid hemorrhage). The mechanisms of postoperative SRC with neurovascular events were assumed to be associated with sympathetic stimulation within the myocardium due to neuroendovascular events [33]. In our study, there were three cases of postoperative SRC (one craniectomy and two embolizations for subarachnoid hemorrhage). However, neurosurgical surgeries or procedures were not independent risk factors in our analysis. There might have been some overlap in diagnosing postoperative SRC with neuro-stress-induced cardiomyopathy.

Similarly, there could have been an overlap when diagnosing postoperative SRC with sepsis-induced cardiomyopathy. Our diagnosis criteria for postoperative SRC regarding sepsis might have overlapped both sepsis-induced cardiomyopathy (left ventricular dilatation with low ejection fraction) and sepsis-induced TTC (typical apical ballooning) [34]. In our study, there were five postoperative sepsis patients in the postoperative SRC group (two patients with sepsis-induced cardiomyopathy and three patients with sepsis-induced TTC). One of the two patients with sepsis-induced cardiomyopathy died, whereas three patients with sepsis-induced TTC patients lived. Although there is a lack of information regarding the prognosis of sepsis-induced cardiomyopathy and TTC, sepsis-induced TTC had favorable outcomes (92.3% alive), which was reported by a recent systemic review similar to our study [35].

Another issue to take note of in our study is the definition of postoperative SRC. There are various methods of diagnosing SRC, including the Mayo Clinic criteria and Johns Hopkins criteria [36], and its prevalence and risk factors can change depending on the diagnostic criteria because of the varying spectrum of SRC [6]. According to the 2007 Japanese guidelines, the development of SRC with cerebrovascular disease or pheochromocytoma was an exclusion criterion for diagnosing TTC. However, because cerebrovascular disease and pheochromocytoma are also important stressful conditions during the perioperative period, we followed other diagnostic criteria such as the Johns Hopkins criteria or Gothenburg criteria [37]. As a result, two patients who underwent embolization for subarachnoid hemorrhage, one patient who underwent craniectomy for intracranial hemorrhage, and one patient who underwent adrenalectomy for pheochromocytoma were included in our study. We diagnosed patients with postoperative SRC only if they had undergone postoperative coronary evaluation. Generally, when diagnosing SRC, a coronary evaluation is suggested to exclude acute coronary syndrome; however, in clinical practice, a coronary evaluation is not always conducted [20]. The strength of our study was that all patients suspected of having postoperative SRC underwent coronary evaluation to exclude acute coronary syndrome to ensure an accurate evaluation of the prevalence because a group of patients with SRC for which acute coronary syndrome and SRC were not definitively separated may have existed as a result of death before the coronary evaluation. Therefore, to estimate the prevalence and risk factors, it was appropriate to exclude the group of patients with SRC from our study.

Our study had limitations. First, due to its retrospective design, bias may have been present. Second, because it was a prevalence study conducted in a single country at a single center, generalizing the results may be problematic. Moreover, SNUBH has excellent health care services, which might have lowered the prevalence of postoperative SRC. We set the postoperative period from after surgery until discharge; therefore, the definition of the postoperative recovery point may be controversial. Finally, as mentioned previously, a group of patients with SRC may have existed; in other words, patients whose condition worsened postoperatively may have had SRC but were excluded because of death before evaluation.

In conclusion, our study found that the prevalence of postoperative SRC was 17.74 patients per 100,000 adult patients over the course of 5 years, with four cases of in-hospital mortality. Factors that increase the risk of postoperative SRC included higher ASA score (≥3), preoperative hyponatremia, and higher preoperative BMI.

## Supporting information

S1 Table Information for postoperative SICMP patients in five years. (DOCX)
