# Supplementary material for: Prevalence and risk factors for postoperative stress-related cardiomyopathy in adults
Source: PLoS One. 2017 Dec 20;12(12):e0190065. doi: 10.1371/journal.pone.0190065 (PMC5738130; doi:10.1371/journal.pone.0190065)
Supplement: S1 Table — (DOCX) [file pone.0190065.s001.docx]

Table S1. Information for postoperative SICMP patients in five years

| Operation | EF  SICMP  (%) | EF after Recovery  (%) | 2D Echo  Finding | Symptom onset  (POD) | Initial Symptom | Troponin I (recovery)  (ng/mL) | Troponin I (Max)  (ng/mL) | APACHE II score | Sepsis  Occurrence | Outcome |
| --- | --- | --- | --- | --- | --- | --- | --- | --- | --- | --- |
| Mean  (SD) | 41.94  (8.29) | 61.49  (5.03) |  | 2.53  (3.87) |  | 0.19  (0.43) | 4.08  (6.07) | 21.59  (9.53) |  |  |
| Urologic Procedure | 42 | 59.38 | Typical Apical Ballooning | 2 | ECG change | 0.035 | 1.97 | 21 | Sepsis | Alive |
| Craniectomy | 38 | 62 | Typical Apical Ballooning | 1 | Shock | 0.096 | 0.423 | 22 | No Sepsis | Alive |
| Urologic Procedure | 40 | 65.03 | Typical Apical Ballooning | 2 | Shock | 0.074 | 2.32 | 32 | Sepsis | Alive |
| PPPD | 35 | 68 | Global hypokinesia | 16 | ECG change | 0.075 | 2.05 | 24 | Sepsis | Alive |
| Lower Limb Surgery | 37 | 59 | Typical Apical Ballooning | 1 | Shock | 0.186 | 6.35 | 15 | No Sepsis | Alive |
| Pulmonologic Procedure | 50 | 65 | Typical Apical Ballooning | 4 | Hypoxia | 0.085 | 26.2 | 16 | Sepsis | Alive |
| Plastic surgery | 40 | 63 | Multi-vessel territory RWMA | 7 | Altered Mentality | 0.092 | 2.35 | 13 | No Sepsis | Alive |
| Gastrectomy | 45 | 60 | Multi-vessel territory RWMA | 0 | Shock | 0.077 | 1.85 | 47 | Sepsis | Death |
| Bowel Resection | 35 | 58 | Typical Apical Ballooning | 1 | ECG change | 0.097 | 0.968 | 12 | No Sepsis | Alive |
| Bowel Resection | 50 | 66 | Typical Apical Ballooning | 0 | Chest pain | 0.035 | 0.7 | 20 | No Sepsis | Alive |
| Bowel Resection | 55 | 65 | Typical Apical Ballooning | 5 | Chest pain | 0.044 | 3.98 | 13 | No Sepsis | Alive |
| ENT surgery | 30 | 45 | Global hypokinesia | 2 | ECG change | 0.051 | 5.95 | 38 | No Sepsis | Death |
| Embolization  (SAH) | 40 | 60 | Multi-vessel territory RWMA | 0 | ECG change | 1.89 | 10 | 26 | No Sepsis | Alive |
| Embolization  (SAH) | 36 | 60 | Typical Apical Ballooning | 1 | Cardiac Arrest | 0.196 | 2.01 | 16 | No Sepsis | Death |
| Lung Resection | 60 | 65 | Typical Apical Ballooning | 0 | Dyspnea | 0.075 | 1.09 | 15 | No Sepsis | Alive |
| Cholecystectomy | 50 | 60 | Typical Apical Ballooning | 1 | Chest pain | 0.04 | 0.076 | 25 | No Sepsis | Alive |
| Adrenalectomy | 30 | 65 | Global hypokinesia | 0 | ECG change | 0.035 | 1.08 | 12 | No Sepsis | Death |
| SICMP, Stress Induced Cardiomyopathy; EF, Ejection Fraction; APACHE, Acute Physiology and Chronic Health Evaluation; PPPD, [Pylorus-Preserving Pancreaticoduodenectomy; RWMA, Regional Wall Mortion Abnormality; ECG, Electrocardiogram; LAD, Left anterior descending; SAH, Subarachnoid hemorrhage](http://emedicine.medscape.com/article/1893199-overview) | | | | | | | | | | |
